# Single-cell RNA-seq reveals RAD51AP1 as a potent mediator of EGFRvIII in human glioblastomas

**DOI:** 10.18632/aging.102282

**Published:** 2019-09-18

**Authors:** Qixue Wang, Yanli Tan, Chuan Fang, Junhu Zhou, Yunfei Wang, Kai Zhao, Weili Jin, Ye Wu, Xiaomin Liu, Xing Liu, Chunsheng Kang

**Affiliations:** 1 Tianjin Medical University General Hospital, Tianjin 300052, China; 2 Tianjin Neurological Institute, Key Laboratory of Neurotrauma, Variation, and Regeneration, Ministry of Education and Tianjin Municipal Government, Tianjin 300052, China; 3 Department of Pathology, Medical College of Hebei University, Baoding, Hebei 071000, China; 4 Department of Neurosurgery, Hebei University Affiliated Hospital, Baoding 071000, China; 5 Radiosurgery Center, Department of Neurosurgery, Tianjin Huanhu Hospital, Nankai University, Tianjin 300350, China; 6 Beijing Neurosurgical Institute, Capital Medical University, Beijing 100050, China; 7 Affiliated Cancer Hospital and Institute of Guangzhou Medical University, Guangzhou 510095, China

**Keywords:** glioblastoma, heterogenous, EGFRvIII, single-cell sequencing, RAD51AP1

## Abstract

Recent advances in single-cell RNA sequencing (scRNA-seq) have endowed researchers with the ability to detect and analyze the transcriptomes of individual cancer cells. In the present study, 16,128 tumor cells from EGFR wild-type and EGFRvIII mutant cells were profiled by scRNA-seq. Analyses of scRNA-seq data from both U87MG and U87MG-EGFRvIII libraries revealed inherent heterogeneity in gene expression and biological processes. The cells stably expressing EGFRvIII showed enhanced transcriptional activities and a relatively homogeneous pattern, which manifested as less diverse distributions, gene expression levels and functional annotations compared with those of cells expressing the nonmutated version. Moreover, the differentially expressed genes between the U87MG and U87MG-EGFRvIII groups were mainly enriched in DNA replication, DNA repair and angiogenesis. We compared scRNA-seq data with bulk RNA-seq and EGFRvIII xenograft RNA-seq data. RAD51AP1 was shown to be upregulated in all three databases. Further analysis of RAD51AP1 revealed that it is an independent prognostic factor of glioma. Knocking down RAD51AP1 significantly inhibited tumor volume in an intracranial EGFRvIII-positive GBM model and prolonged survival time. Collectively, our microfluidic-based scRNA-seq driven by a single genetic event revealed a previously unappreciated implication of EGFRvIII in the heterogeneity of GBM and identified RAD51AP1 as an oncogene in glioma.

## INTRODUCTION

Glioblastoma (GBM; World Health Organization grade IV) is the most common and devastating primary tumor in the central nervous system [1, 2]. Despite multimodal treatments involving surgery, radio- and chemotherapy, patients with GBM have an average survival time of only slightly more than one year [1, 2]. Extensive investigations have suggested that the dismal prognosis of GBM is largely attributed to inevitable therapeutic resistance and tumor relapse, while heterogeneity has been described as the root cause of multiple cancer types [3, 4]. Therefore, an improved understanding of GBM heterogeneity has important implications for not only clinical diagnoses but also for the design of better therapies and avoidance of tumor recurrence [5, 6].

Previous efforts with a focus on bulk tissue have revealed a remarkably heterogeneous pattern among individual patients. Receptor tyrosine kinases (RTKs), especially the epidermal growth factor receptor (EGFR), are crucial regulators of cellular proliferation, angiogenesis, metabolism and survival [7, 8]. Importantly, the deletion of exons 2-7 of EGFR (EGFRvIII) is a common genetic alteration, accounting for nearly 30% of GBM cases [8]. EGFRvIII, which lacks the extracellular ligand-binding domain, could constitutively activate the EGFR signaling pathway, leading to the malignant progression of tumor cells and modulation of the tumor microenvironment [9]. While this alteration can drive gliomagenesis, tumors harboring EGFRvIII are heterogeneous [10, 11]. However, conventional methods failed to adequately reflect intratumoral composition.

DNA damage is a high risk factor that leads to replication errors, cell cycle arrest, cell death and human disease. RAD51-mediated homologous recombination is an important method for repairing DNA double-stranded breaks (DSBs). RAD51AP1 (RAD51-associated protein 1), first identified as a RAD51-interacting protein [12], stimulates joint molecule formation and is required for cellular protection against DSB-inducing agents [13, 14]. Because of the importance of DSBs, chemotherapies that induce DSBs are widely employed in cancer treatment. Thus, molecules involved in DSB repair could influence chemotherapeutic drug effectiveness [15]. Although reported in ovarian cancer, lung cancer and melanoma, RAD51AP1 is still a rarely studied protein [16, 17], and its role in glioma is unknown.

The development of single-cell RNA sequencing (scRNA-seq) techniques has enabled transcriptomic analysis within individual cells. Using scRNA-seq libraries, an increasing number of studies have attempted to dissect lineage identity, tracking dynamic cellular changes and depicting the interplay between intrinsic tumor cells and the microenvironment, thereby uncovering the intratumoral heterogeneity in glioma [18–20]. In the present study, we used microfluidic-based scRNA-seq techniques to profile single cells from U87MG and EGFRvIII-expressing U87MG cell lines, which we found to exhibit inherently variable gene expression and biological functions. We also observed enhanced transcriptional activity and decreased heterogeneity caused by the EGFRvIII mutation. By comparing the two scRNA-seq libraries, we showed that EGFRvIII could induce a phenotypic transition to enhance DNA division, DNA repair and angiogenesis. We combined the scRNA-seq data with bulk U87/U87-EGFRvIII RNA-seq data and EGFRvIII xenograft RNA-seq data under accession number GSE46028 and found RAD51AP1 to be upregulated in three of the databases. Furthermore, we showed that RAD51AP1 was a GBM oncogene by bioinformatics analysis, generated an intracranial mouse glioma model and performed clinical multiple spot samplings. Therefore, our study reveals the impact of EGFRvIII on the dynamic alterations of glioma cells at single-cell resolution, further elucidating the exact mechanism of EGFRvIII in glioma and identifying the role of RAD51AP1 in GBM.

## RESULTS

### scRNA-seq analysis of U87MG and U87MG-EGFRvIII cells

U87MG is a GBM cell line that is widely used in experimental investigations. To evaluate the effects of EGFRvIII mutation on GBM, U87MG cells were transfected with lentivirus containing EGFRvIII cDNA. Then, U87MG and U87MG-EGFRvIII cells were subjected to scRNA-seq analysis using microfluidic-based approaches with the 10x Genomics® platform [21]. A total of 9,365 cells and 20,033 UMIs per cell were estimated to exist in the U87MG library (Table 1). Although the number of loaded cells was less than that in the U87MG library (6763 cells), the median UMI counts and genes per cell were 26,811 and 4238, respectively, in the U87MG-EGFRvIII library, and these numbers were higher those in its counterpart (Table 1). This augmentation of UMI counts and genes within individual cells was indicative of a reinforcement of EGFRvIII on whole-genome transcriptomic activities.

**Table 1:** Summary of 10 × Genomics Single-cell RNA Sequencing.

**Summary of barcodes and sequencing parameters**	**U87MG**	**U87MG-EGFRvIII**
Estimated number of cells	9,365	6,763
Fraction reads in cells	80.9%	75.9%
Mean reads per cell	56,095	92,544
Median genes per cell	3,890	4,238
Total genes detected	16,094	15,874
Median UMI counts per cell	20,033	26,811
Reads mapped confidently to the transcriptome	66.6%	69.5%
Reads mapped confidently to exonic regions	70.8%	73.5%
Reads mapped confidently to intronic regions	13.2%	10.5%
Reads mapped confidently to intergenic regions	5.8%	4.5%
Reads mapped antisense to the gene	5.2%	5.0%

**Figure 1:**
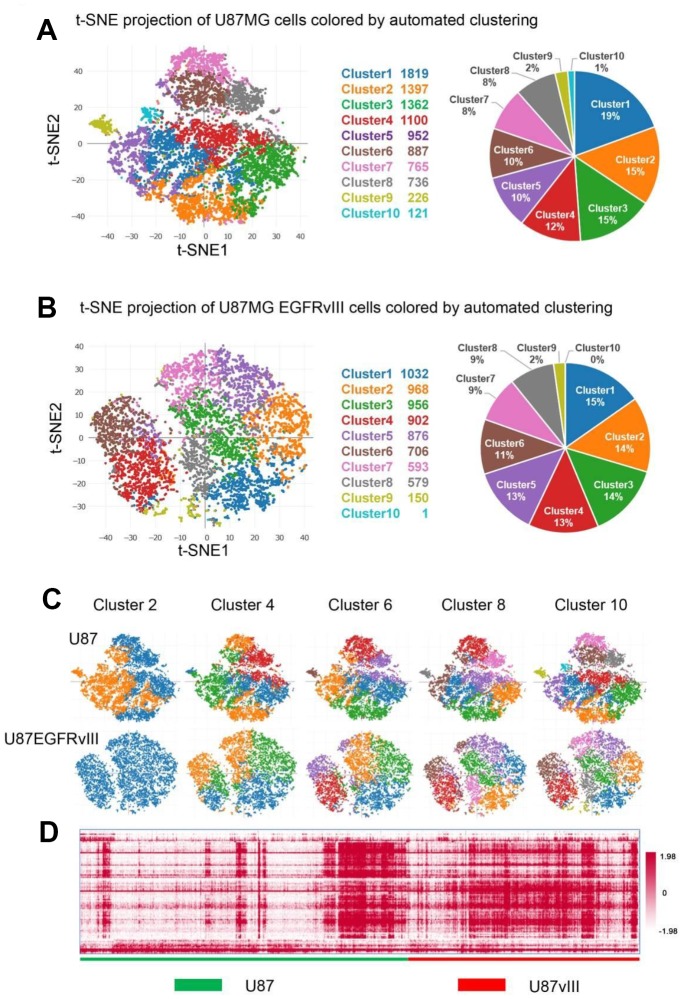
**Single-cell analyses of U87MG and U87MG-EGFRvIII cells. U87MG-EGFRvIII cells were less heterogeneous than U87MG cells.** (**A**) Clustering analyses reveal ten subsets with cluster-specific genes and functions. The pie chart shows the percentage of each cluster. (**B**) The clustering results of U87MG-EGFRvIII cells (k=10) and the percentage of each cluster. (**C**) The clustering results with k values from two to ten. (**D**) The heatmap shows the gene expression of every single cell.

Next, K-means clustering analysis was conducted to examine cellular heterogeneity. Overall, ten distinct cell clusters were identified and visualized by the two-dimensional projection of t-distributed stochastic neighbor embedding (t-SNE, Figure 1A and 1B) [22]. Interestingly, when k was equal to two, the majority of U87MG-EGFRvIII cells belonged to the same cluster (Figure 1C). In addition, automated clustering revealed an attenuated heterogeneity of U87MG-EGFRvIII at k=4, 6, 8 and 10 (Figure 1C). The top 100 differential expression genes (Supplementary Table 1) were picked up, and clustered in all the 16128 cells. From the heatmap we can see that U87-EGFRvIII cells are more homogeneous than U87 cells (Figure 1D).

The percentage of each cluster ranged from 1% to 19%, and the top three clusters occupied a 49% proportion in U87MG cells. GO analyses of the cluster-specific genes revealed distinct biological subtypes. Briefly, clusters two and seven were enriched in DNA repair, the cell cycle and DNA replication; cluster ten was enriched in immune and inflammatory responses; and clusters four, six and eight were associated with cell adhesion and angiogenesis (Supplementary Figure 1). Notably, clusters one, three and nine showed too few differentially regulated genes to perform GO analysis. For U87MG-EGFRvIII cells, only clusters 1, 4, 6 and 8 showed enough cluster-specific genes for GO analyses (Supplementary Figure 2).

### Comparison of U87MG and U87MG-EGFRvIII scRNA-seq

**Figure 2:**
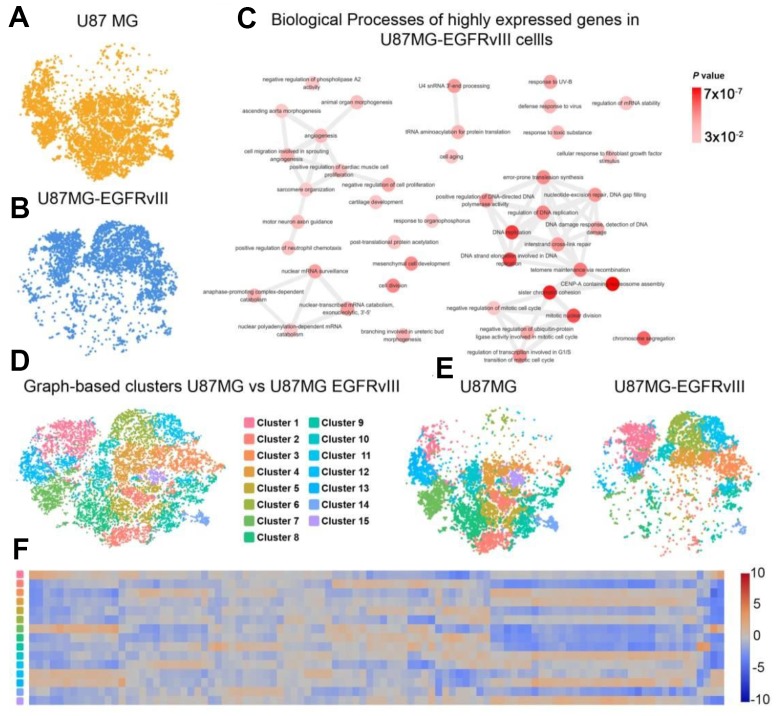
**Comparison of single-cell libraries from U87MG and U87MG-EGFRvIII cells.** (**A**) The distribution of U87MG cells. (**B**) The distribution of U87MG-EGFRvIII cells. (**C**) The biological process annotations of differential genes that were upregulated in EGFRvIII cells. (**D**) Graph-based clustering revealed 15 clusters in 16,128 cells. (**E**) Distributions of each cluster in the U87MG and U87MG-EGFRvIII libraries. (**F**) The expression levels of cluster-specific genes.

**Figure 3:**
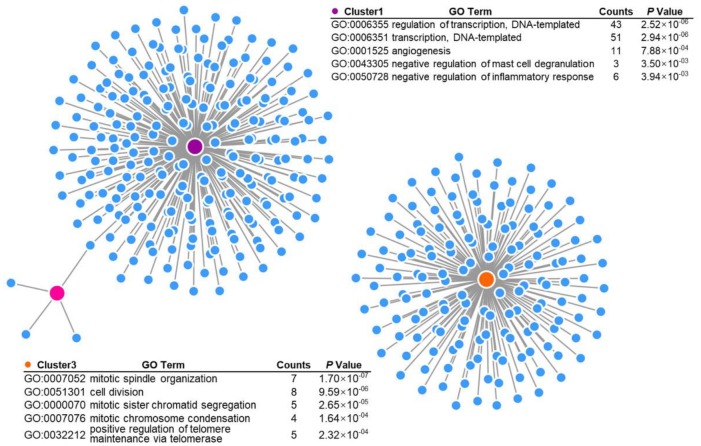
**Gene Ontology (GO) analysis of EGFRvIII-related cluster-specific genes and biological processes (cluster 1, cluster 3, and cluster 6).**

To further explore the transcriptomic differences between U87MG and U87MG-EGFRvIII cells, we subjected both libraries to the LOUPE browser simultaneously. As expected, the two populations of cells showed distinct distribution patterns (Figure 2A, 2B). The upregulated genes in U87MG-EGFRvIII cells were mainly enriched in the DNA damage response, cell division and angiogenesis processes, which was consistent with the TCGA results (Figure 2C and Supplementary Figure 2). Graph-based clustering analysis further identified 15 different subgroups with cluster-specific genes and biological processes (Figure 2D–2F). The U87MG-EGFRvIII cells were mainly distributed in cluster one (1397, 71.42%), cluster three (1108, 74.11%), cluster six (1173, 91.00%) and cluster eleven (775, 96.75%), while U87MG cells were observed in the other eleven clusters (Supplementary Figure 3), which further indicated that the heterogeneity is stronger in U87MG cells. Consistently, the biological annotations revealed that the cluster-specific biological processes in U87MG-EGFRvIII cells are associated with angiogenesis (cluster 1), DNA repair and cell division (Figure 3).

### RAD51AP1 is closely correlated with EGFRvIII

**Figure 4:**
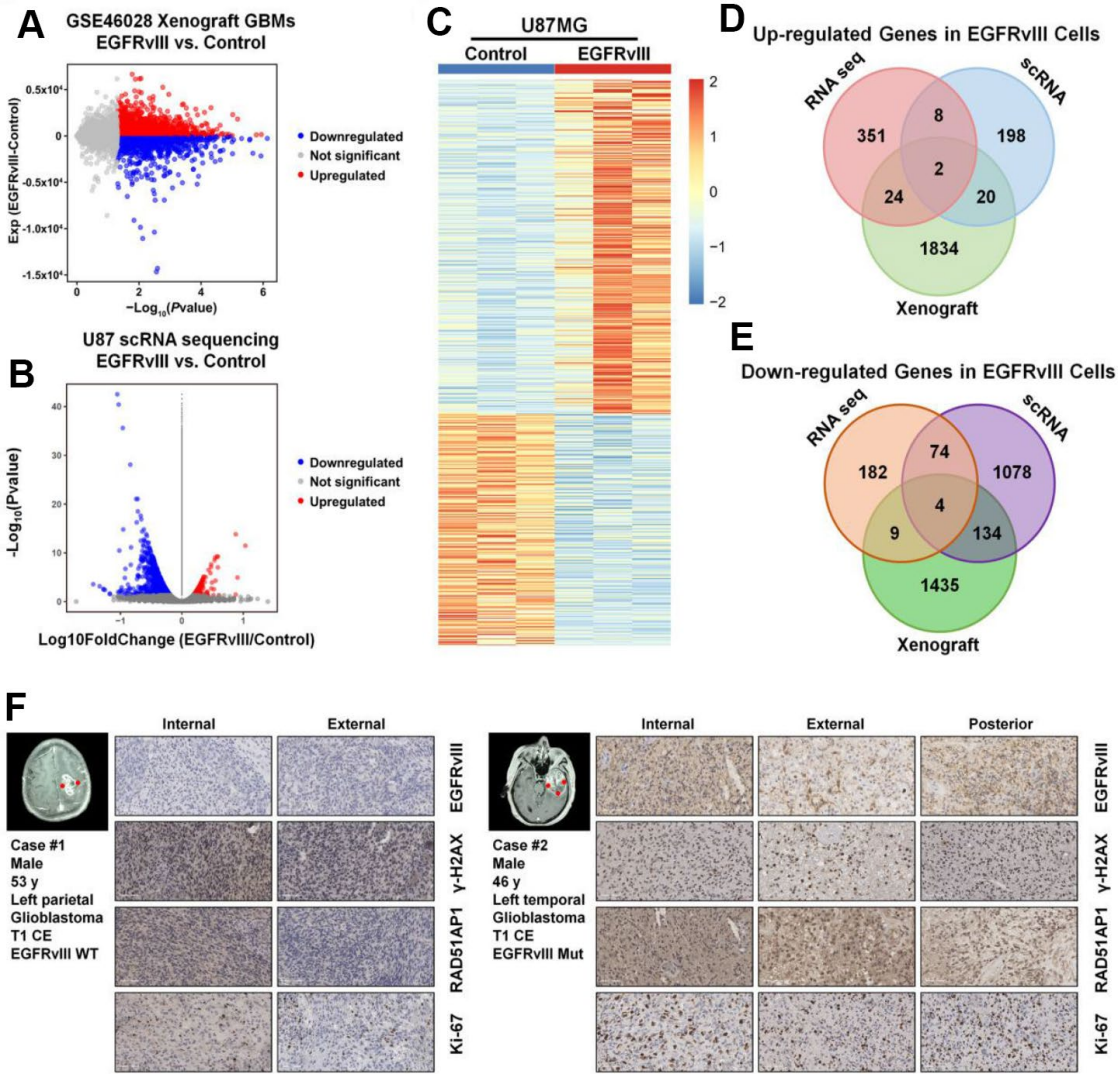
**RAD51AP1 is upregulated in EGFRvIII-positive cells.** The volcano plot was constructed to profile the differentially expressed genes observed in GES46028 (**A**) and scRNA-seq data (**B**). (**C**) A heatmap was employed to profile the differentially expressed genes observed in U87MG/U87MG-EGFRvIII RNA-seq data. A Venn diagram was used to profile the common upregulated (**D**) and downregulated (**E**) genes in three databases. (**F**) The EGFRvIII, r-H2A.x, RAD51AP1 and Ki-67 expression levels in multipoint samples from two patients were examined by IHC staining.

To further profile the differential genes associated with EGFRvIII expression, we employed GSE46028, a xenograft GBM RNA-seq database, and the RNA-seq results of U87MG vs U87MG-EGFRvIII cells. In total, 1880 upregulated genes and 1582 downregulated genes were observed in the EGFRvIII group in the GSE46028 database (Figure 4A), 228 upregulated genes and 1290 downregulated genes were observed in EGFRvIII-positive cells (Figure 4B), and 385 upregulated genes and 269 downregulated genes were observed in the RNA-seq results (Figure 4C). We combined these data and found that two upregulated genes and four downregulated genes coincided in the three datasets (Figure 4D, 4E). Among them, RAD51AP1 was positively correlated with the EGFRvIII mutation. Moreover, both the protein expression of RAD51AP1 and the proliferative index Ki-67 were higher in EGFRvIII mutant specimens than in EGFR wild-type samples (Figure 4F).

### The oncogenic role of RAD51AP1 in GBM

**Figure 5:**
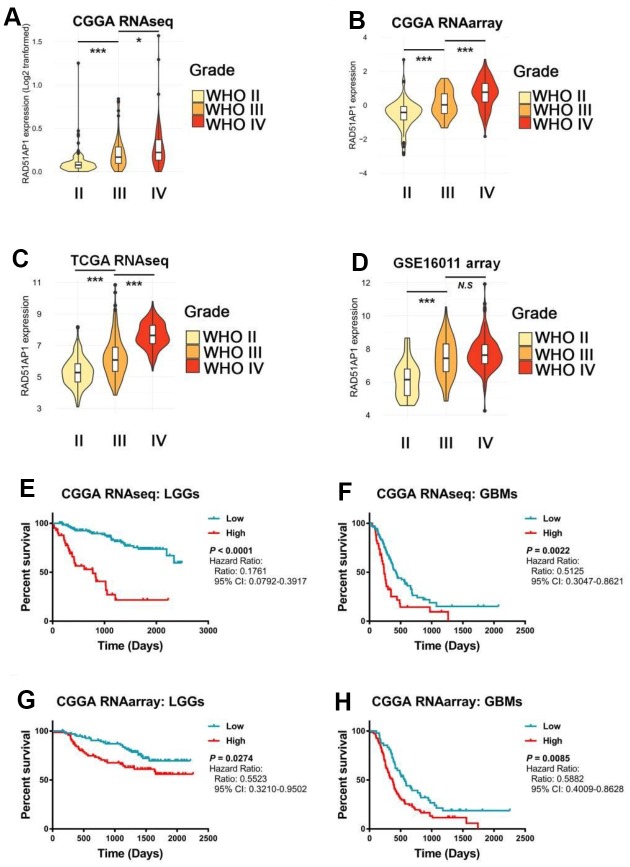
**The expression level of RAD51AP1 correlated with the GBM clinical grade and patient survival rate.** (**A**–**D**) ssGSEA was employed to evaluate the expression pattern of RAD51AP1 in the CGGA, TCGA and GSE16011 databases. (**E**–**H**) Kaplan-Meier survival curves were plotted to show the survival times at different RAD51AP1 expression levels.

**Figure 6:**
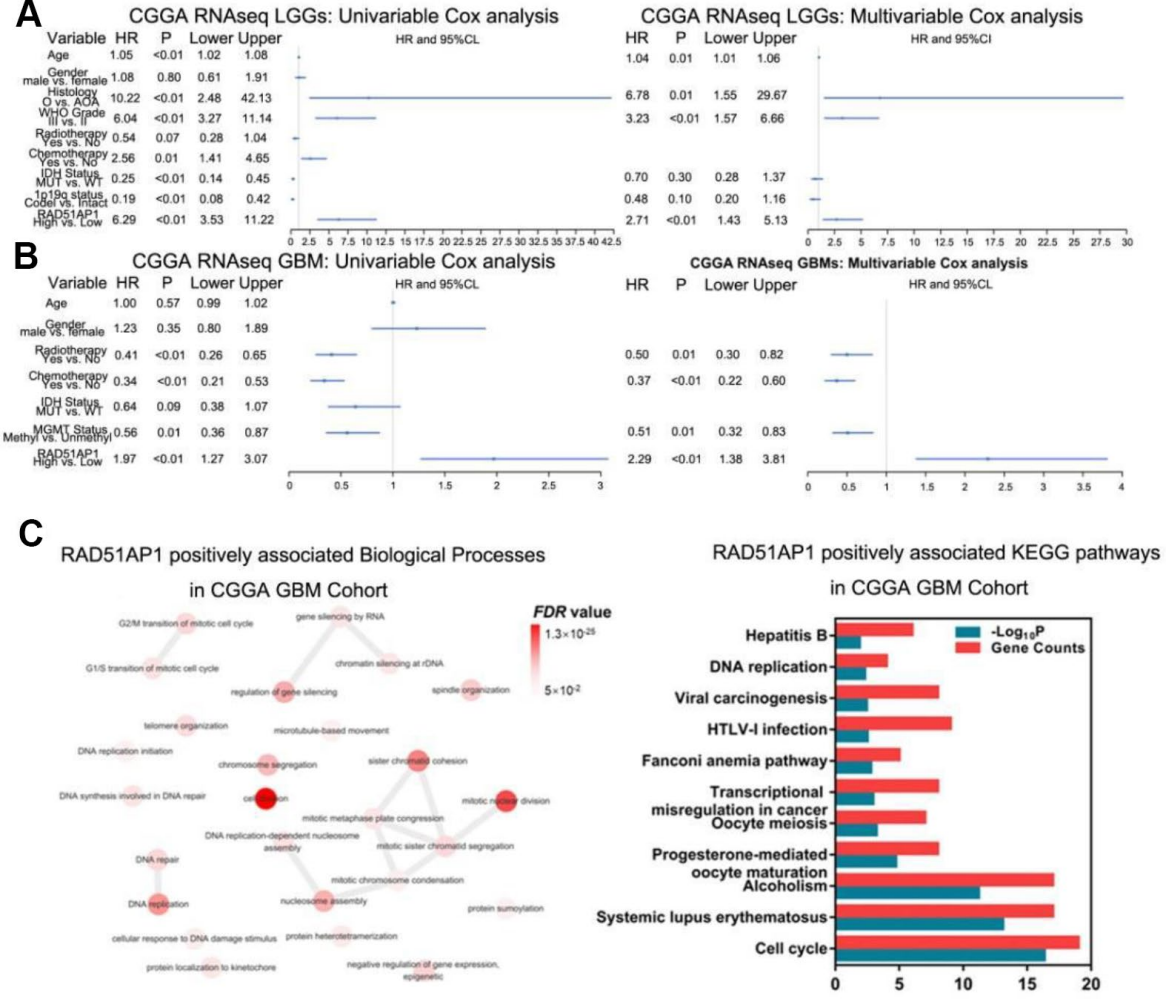
**RAD51AP1 is an oncogene in glioma.** (**A**) RAD51AP1 highly coincides with EGFRvIII in scRNA-seq data. (**B**) GSEA was performed to estimate RAD51AP1 expression in gliomas of different clinical grades. (**C**) Uni- and multivariable Cox analyses were performed to evaluate the role of RAD51AP1 in gliomas in the CGGA database, while GO and KEGG analyses were employed to profile the pathways of RAD51AP1-related genes in the CGGA database.

While RAD51AP1 is known to promote RAD51-mediated homologous recombination [13], the role of RAD51AP1 in glioma has rarely been studied. Bioinformatic analyses revealed that RAD51AP1 is significantly enriched in high-grade gliomas in the CGGA, TCGA and GSE16011 datasets (Figure 5A–5D). Kaplan-Meier survival analysis confirmed the poor outcomes of patients with both low- and high-grade gliomas expressing high levels of RAD51AP1 (Figure 5E–5H). Uni- and multivariable Cox analyses further indicated that RAD51AP1 is an independent prognostic factor of clinical and molecular pathological parameters in the CGGA and TCGA databases (Figure 6A and 6B and Supplementary Figure 4). Notably, the RAD51AP1 positively associated genes were mainly enriched in DNA repair and cell cycle-related biological processes and KEGG pathways in both the CGGA and TCGA databases (Figure 6C and Supplementary Figure 5).

**Figure 7:**
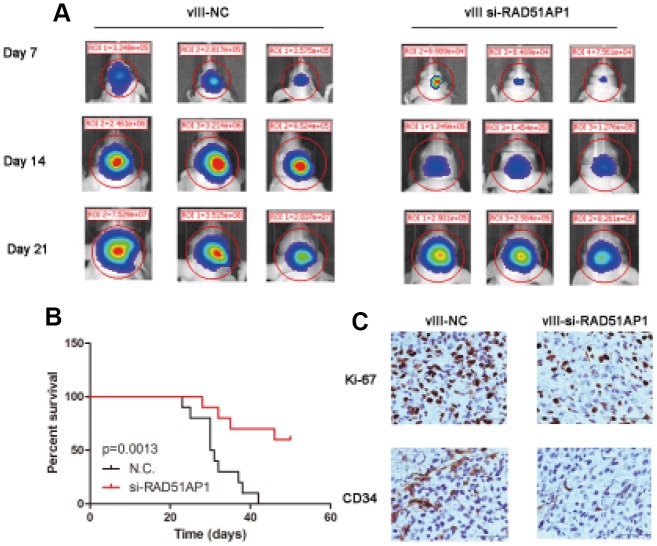
**Target knocking down RAD51AP1 inhibited the progression of the EGFRvIII-positive intracranial GBM model.** (**A**) The tumor volumes at the indicated times were evaluated by bioluminescence imaging. (**B**) Survival rates of mice bearing U87-EGFRvIII and EGFRvIII-siRAD51AP1 tumors. (**C**) Immunohistochemistry analysis was performed to detect Ki-67 and CD34 expression.

To investigate the role of RAD51AP1, we constructed intracranial mouse models using U87MG-EGFRvIII cells transfected with a negative control (N.C.) or si-RAD51AP1 lentivirus. *In vivo* imaging analysis at days 7, 14 and 21 revealed that knocking down RAD51AP1 significantly inhibited the tumor volume compared to that in the Lenti-N.C. group (Figure 7A). Low RAD51AP1 expression was strongly associated with longer survival times in mice (Figure 7B). Furthermore, immunohistochemistry (IHC) analysis indicated that the CD34 and Ki-67 expression levels were reduced in the RAD51AP1 knockdown group (Figure 7C).

Altogether, these results demonstrated that RAD51AP1 is an oncogene in glioma and is highly associated with EGFRvIII.

## DISCUSSION

GBM is a complex entity composed of cells with various phenotypes and genotypes. Molecular profiling of bulk tumor tissues suggests an intertumoral diversity by dividing patients into discrete subpopulations [23, 32], while multiregional sampling and single-cell sequencing reveal the spatial heterogeneity within individual tumors [24, 25]. Uncovering this heterogeneity in GBM will provide better insights into the mechanism underlying tumor cell behavior. In the present study, U87MG cells, a widely used experimental cell line, exhibited varied gene transcript levels and several biological processes. For instance, clusters two and seven, which accounted for 23% of all the detected cells, were enriched in cell cycle transition, DNA replication, cell division and DNA repair, thus potentially contributing to the variation in therapeutic responses of tumor cells observed under the same conditions.

Our preliminary investigations shown that mutations in EGFRvIII affect exosome formation, proliferation, the cytoskeleton and several malignant cellular processes by transcriptional, posttranscriptional and epigenetic regulatory mechanisms [26–30]. Based on these works, we attempted to further map the EGFRvIII-induced cell identity from single-cell gene expression profiles. ScRNA-seq analysis of EGFRvIII mutant cells demonstrated increased numbers of mean reads, mean genes and median UMI counts per cell, indicating a higher transcriptional activity conferred by EGFRvIII than by the nonmutated version. This result is consistent with pioneering investigations demonstrating that EGFR mutation remodels the activated enhancer landscape through epigenetic reprogramming by promoting GBM tumorigenesis *in vitro* and *in vivo* [31]. Although the crosstalk between malignant and nonmalignant GBM cells is complex and heterogeneous, the tumor cells driven by the EGFRvIII mutation showed a relatively uniform pattern of distribution and gene expression. The biology of EGFRvIII has been studied extensively, and EGFRvIII has been shown to be a strong oncogene that can drive a more aggressive phenotype. Moreover, the cells stably expressing EGFRvIII showed enhanced malignancies of angiogenesis, DNA repair and DNA replication, which verified the consequence proposed by bulk tissue sequencing and experimental investigations [10, 11].

To the best of our knowledge, we herein identify RAD51AP1 as an oncogene in glioma for the first time. Temozolomide (TMZ) is the only chemotherapeutic drug used to treat GBM and functions by inducing DNA damage in tumor cells. Unfortunately, the average survival time of glioma patients is only 15 months because patients become resistant to the drug TMZ [1]. Thus, our work provides a new possibility of combining TMZ and RAD51AP1, which might enhance the DNA damaging effect of TMZ.

In conclusion, heterogeneity poses a substantial challenge to the treatment of glioma patients. Using scRNA-seq and quantitative methods, we delineated transcriptomic and functional variations in U87MG and U87MG-EGFRvIII cells, emphasized the importance of EGFRvIII mutations for tumor cell aggressive behavior and heterogeneity, and identified RAD51AP1 as an oncogene in glioma for the first time.

## MATERIALS AND METHODS

### Cell culture and lentivirus infection

The human GBM cell line U87MG was obtained from American Type Culture Collection (ATCC). The cells were cultured in complete DMEM supplemented with 100 units/ml penicillin and 50 μg/ml streptomycin. Lentivirus containing EGFRvIII cDNA was purchased from GENECHEM (Shanghai, China), and cells were infected with the virus according to the manufacturer’s instructions. U87MG cells (U87MG-EGFRvIII cells) that stably expressed EGFRvIII were used for subsequent experiments.

### RNA sequence analysis

Clinical characteristics, transcriptome sequencing data and molecular data were downloaded from The Cancer Genome Atlas (TCGA) as a validation set (https://cancergenome.nih.gov/) [32]. Differentially expressed genes were screened using a significant analysis of microarray (SAM) algorithm. Genes with an adjusted *P* value < 0.05 were regarded as candidate differential genes and subjected to Gene Ontology (GO) analysis using the online tool Database for Annotation, Visualization, and Integrated Discovery (DAVID, https://david.ncifcrf.gov/) [33]. The GO results were visualized with the BiNGO plugin imbedded in Cytoscape software (version 3.7.1).

### Single-cell RNA-seq

Briefly, cells were trypsinized and resuspended in a phosphate buffer solution containing 0.04% weight/volume bovine serum albumin (BSA). Barcoded single-cell gel beads in emulsion (GEMs) were created by 10x Genomics® Chromium^TM^ and then reverse transcribed to generate single-cell RNA-seq libraries. Unique molecular identifiers (UMIs), which were incorporated into the 5′ end of cDNA during reverse transcription, were used to quantify the exact number of transcripts in a cell. The single-cell transcriptome analyses were conducted using the LOUPE cell browser. To identify cluster-specific genes, we calculated the expression difference of each gene between that cluster and the average of the rest of clusters. The candidate genes with a fold change > 2 and an adjusted *P* value < 0.05 were used for DAVID analyses.

### Intracranial mouse model

Five-week-old female nude mice were purchased from the Chinese Academy of Medical Science Cancer Institute and randomly divided into two groups. U87-EGFRvIII and U87-EGFRvIII si-RAD51AP1 cells were prepared. A total of 500,000 cells were injected into each mouse under the guidance of a stereotactic instrument. Intracranial tumor growth was measured by bioluminescence imaging on days 7, 14 and 21, and Kaplan-Meier survival curves were plotted to show the survival time.

### Immunohistochemical staining

Immunohistochemistry was performed on mouse intracranial tumors by subjecting 5-μm paraffin sections to a three-step process and a DAB staining kit (ZSGB-BIO). Ki-67 and CD34 primary antibodies were purchased from ZSGB-BIO.

## Supplementary Material

Supplementary Figures

Supplementary Table 1
