# Supplementary material for: Single-cell RNA-seq reveals RAD51AP1 as a potent mediator of EGFRvIII in human glioblastomas
Source: Aging (Albany NY). 2019 Sep 18;11(18):7707–22. doi: 10.18632/aging.102282 (PMC6781999; doi:10.18632/aging.102282)
Supplement: Supplementary Figures [file aging-11-102282-s002.pdf]

SUPPLEMENTARY FIGURES

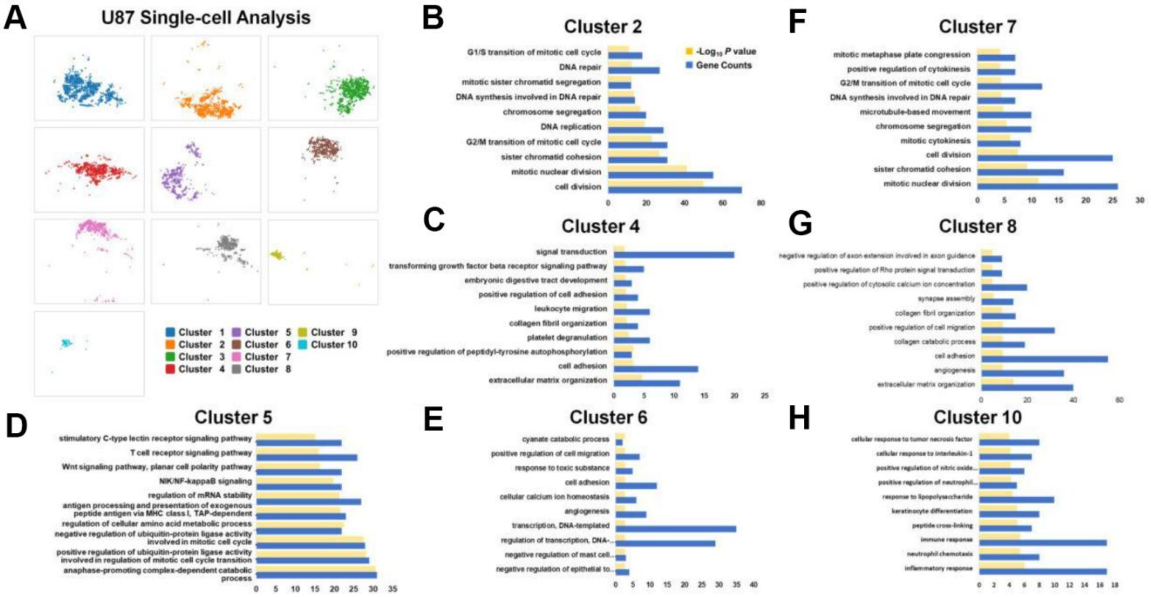

Supplementary Figure 1. Gene Ontology (GO) analysis of each subset using cluster-specific genes in U87MG cells.

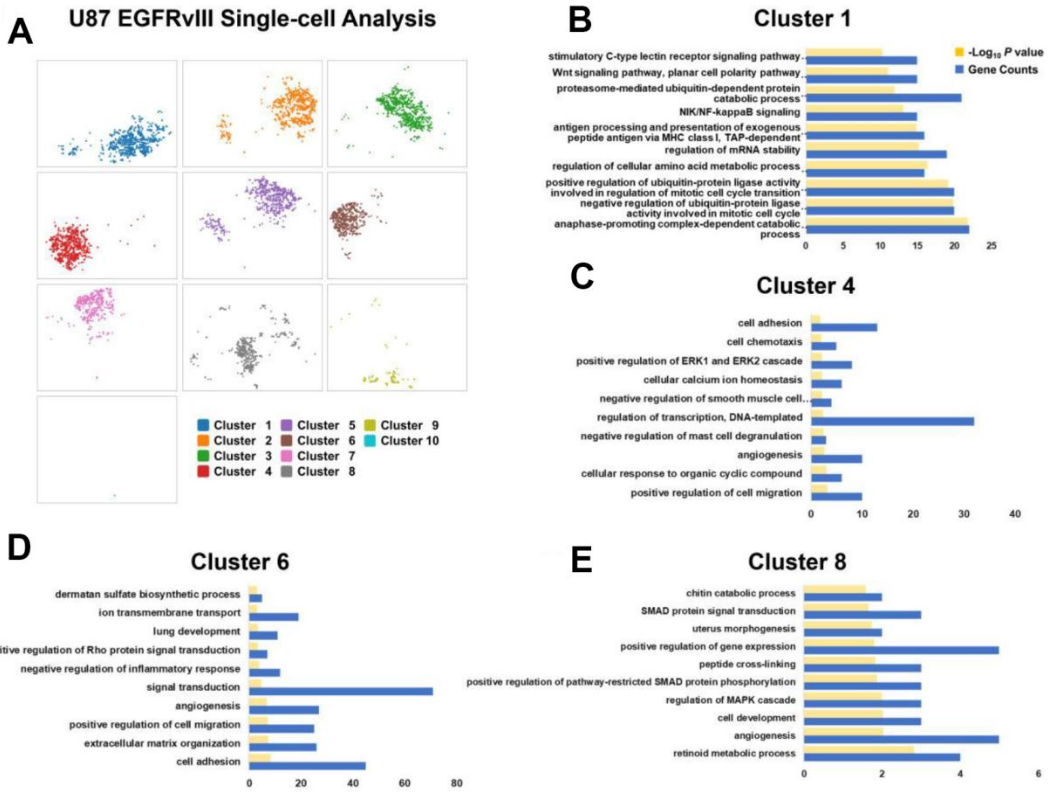

Supplementary Figure 2. Gene Ontology (GO) analysis of each subset using cluster-specific genes in U87MG-EGFRvIII cells.

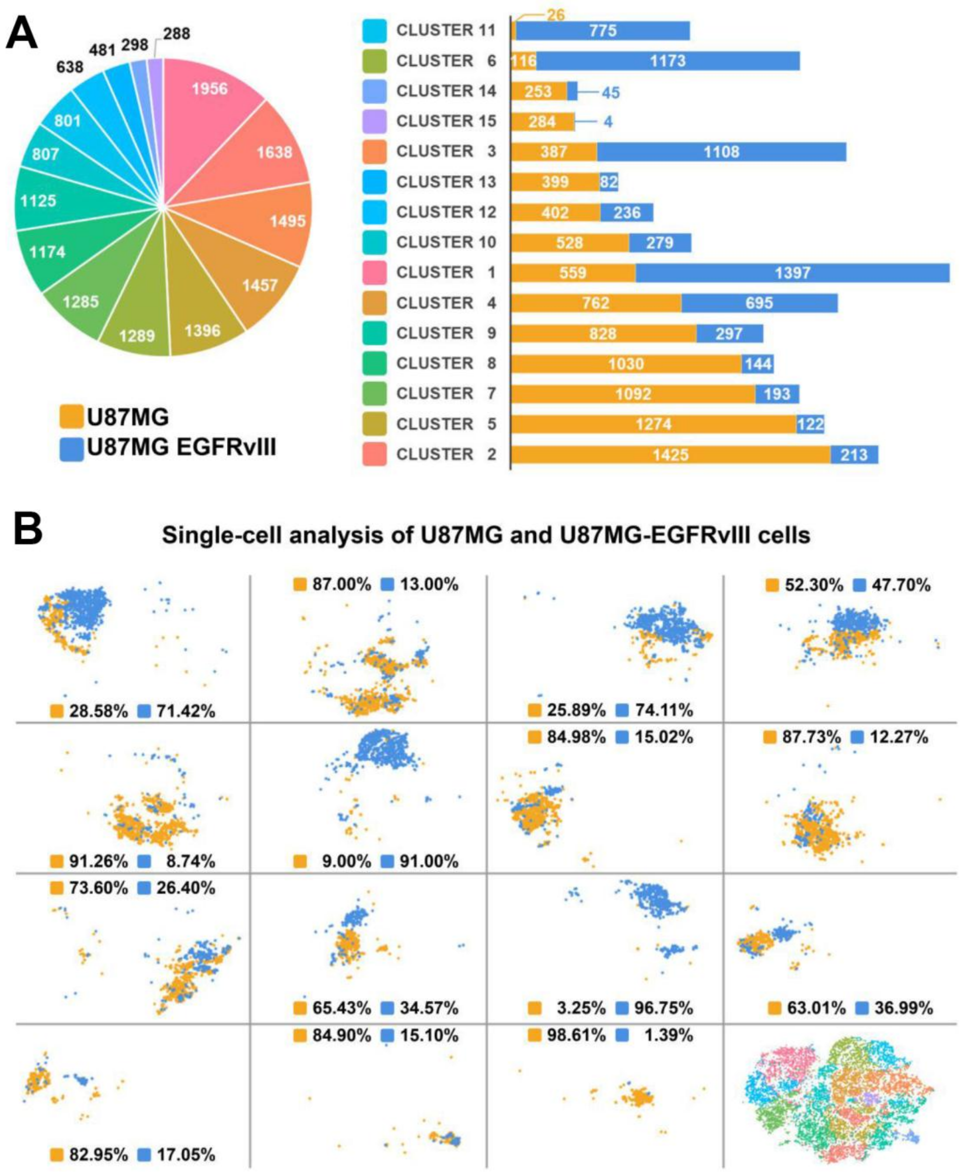

Supplementary Figure 3. The distributions and percentages of U87MG and U87MG-EGFRvIII cells in each cluster.

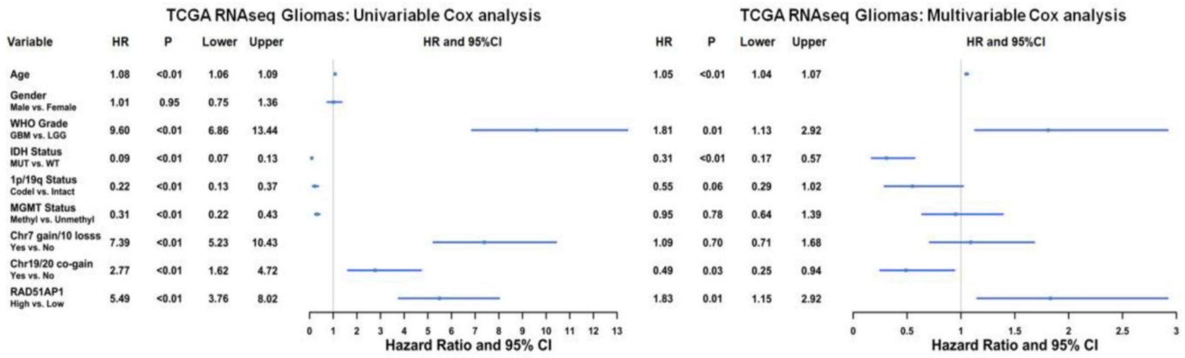

Supplementary Figure 4. Uni- and multivariable Cox analyses were performed to evaluate the role of RAD51AP1 in gliomas in the TCGA database.

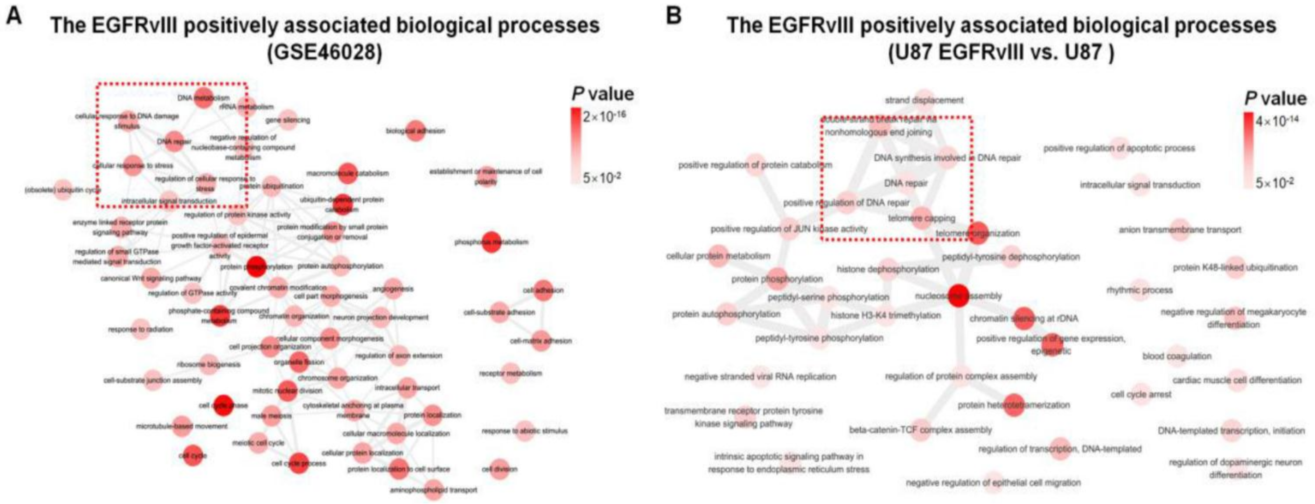

Supplementary Figure 5. GO and KEGG analyses were employed to profile the pathways of RAD51AP1-related genes in the CGGA database.
